# Ameliorative Effect of Curcumin Nanoparticles against Monosodium Iodoacetate-Induced Knee Osteoarthritis in Rats

**DOI:** 10.1155/2022/8353472

**Published:** 2022-10-14

**Authors:** Hadeer M. Hamdalla, Rasha R. Ahmed, Sanaa R. Galaly, Ibrahim A. Naguib, Badrah S. Alghamdi, Osama M. Ahmed, Ahmed Farghali, Manal Abdul-Hamid

**Affiliations:** ^1^Cell Biology, Histology and Genetics Division, Department of Zoology, Faculty of Science, Beni-Suef University, P.O. Box 62521, Beni-Suef, Egypt; ^2^Department of Pharmaceutical Chemistry, College of Pharmacy, Taif University, P.O. Box 11099, Taif 21944, Saudi Arabia; ^3^Department of Physiology, Neuroscience Unit, Faculty of Medicine, King Abdulaziz University, Jeddah 22252, Saudi Arabia; ^4^Pre-Clinical Research Unit, King Fahd Medical Research Center, King Abdulaziz University, Jeddah, Saudi Arabia; ^5^Physiology Division, Zoology Department, Faculty of Science, Beni-Suef University, P.O. Box 62521, Beni-Suef, Egypt; ^6^Department of Material Science and Nanotechnology, Faculty of Postgraduate Studies for Advanced Science, Beni-Suef University, Egypt

## Abstract

**Aim:**

This study is aimed at evaluating the use of curcumin-loaded polylactic-co-glycolic acid nanoparticles (CUR-loaded PLGA NPs) as a treatment against monosodium iodoacetate- (MIA-) induced knee OA.

**Materials and Methods:**

Eighteen rats were assigned to three groups (*n* = 6), namely, normal control group that received intra-articular injections (IAIs) of saline, an OA control group that received an IAIs of MIA (2 mg/50 *μ*L), and a treatment group (MIA+CUR-loaded PLGA NPs) that received IAIs of CUR-loaded PLGA NPs (200 mg/kg b.wt).

**Results:**

The CUR NP treatment against knee OA alleviated radiographic alternations and histopathological changes and inhibited the upregulation in the serum levels of interleukin-1*β*, tumor necrosis factor-*α*, interleukin-6, and transforming growth factor-beta and the downregulation in interleukin-10. CUR NP-treated joints also decreased the mRNA expression of nuclear factor-kappa B and inducible nitric oxide synthase and the protein expression of matrix metalloproteinase-13 and caspase-3. Finally, CUR-loaded PLGA NP treatment mitigated the loss of type II collagen, which resulted in a significant reduction in malondialdehyde level and increased the glutathione content and superoxide dismutase activity compared with that of the OA group.

**Conclusion:**

This study demonstrated that the administration of CUR NPs could provide effective protection against MIA-induced OA and knee joint histological deteriorated changes due to its anti-inflammatory, antioxidant, and antiapoptotic properties.

## 1. Introduction

Osteoarthritis (OA) is a progressive and degenerative illness that happens in the whole joint and can result in articular cartilage degeneration, subchondral bone thickening, synovium inflammation, osteophyte formation, and meniscal degeneration [1]. Treatments for OA are mainly divided into three categories: nonpharmacological treatments, pharmacological treatments limited to analgesics and/or nonsteroidal anti-inflammatory drugs (NSAIDs), and surgical treatments [2]. Current available medications for OA, except joint replacement surgery, are essentially palliative and cannot hinder articular cartilage degradation [3]. Furthermore, long-term use of NSAIDs can cause adverse gastrointestinal, renal, and cardiovascular effects [4]. This calls for the development of a structural OA disease drug that is safe, provides symptomatic relief, and hinders the progression of cartilage degeneration.

Curcumin (CUR) (diferuloylmethane), a polyphenol compound obtained from turmeric, is a naturally available molecule that has robust anticatabolic, antioxidant, anti-inflammatory, and antirheumatic properties [5–7]. Accordingly, CUR seems to be a promising approach in OA therapy. Nevertheless, the therapeutic efficiency of CUR is extremely restricted because of its low water solubility and limited oral bioavailability [8]. Nevertheless, studies have reported that CUR's biological activity could be efficiently enhanced using nanotechnology-based drug delivery [9, 10]. Various studies have explored CUR nanoparticles (NPs) and CUR encapsulation with various substances such as liposomes and polymers to overcome its inherent drawbacks [11, 12]. Polylactic-co-glycolic acid (PLGA) is considered as an effective biodegradable polymeric NPs that was authorized by US Food and Drug Administration for use in drug delivery systems due to its low toxicity, controlled and sustained-release properties, and biocompatibility with tissue and cells [13, 14].

On the other hand, the intra-articular route for drug delivery has significant potency and fewer systemic side effects compared to that of oral delivery [15]. Accordingly, this study was designed to explore the possible feasibility of the intra-articular injections (IAIs) of CUR-loaded PLGA NPs to treat monosodium iodoacetate- (MIA-) induced knee OA in a rat model.

## 2. Materials and Methods

### 2.1. Animals

This study used mature male Wistar rats weighing 130–150 g, which were kept in a standard 12 : 12 light/dark cycle in well-ventilated rooms. One week before initiating the experiments, the rats were housed in sterilized cages to be adapted to the laboratory with free access to water and pellets. All methods for handling, use, and euthanasia of the animals in this study were certified by the Experimental Animal Ethics Committee of Faculty of Science, Beni-Suef University, Egypt, and the ethical authorization number is BSU/FS/2018/15.

### 2.2. Induction of OA

MIA was obtained from Sigma-Aldrich (St. Louis, MO, USA) and dispersed in sterile saline. Under diethyl ether anesthesia, all rats, except the normal control group, received a single intra-articular injection of 50 *μ*L of saline containing MIA (2 mg) into the left side knee joint, as previously described by Ragab et al. [16].

### 2.3. Preparation of CUR-Loaded PLGA NPs

PLGA, which is poly (D, L-lactide-co-glycolide) with lactide: glycolide 50 : 50, molecular weight (24,000), inherent viscosity (1.13 dL/g), and formula [C_3_H_4_O_2_]_x_[C_2_H_2_O_2_]y, was obtained from Sigma-Aldrich (St. Louis, MO, USA). Besides, curcumin, chloroform, polyvinyl alcohol (PVA; MW 30,000–70,000), and ethanol were all purchased from Sigma-Aldrich (St. Louis, MO, USA) as well.

CUR-loaded PLGA NPs were synthesized by solvent solid-in-oil-in-water emulsion (s/o/w) evaporation based on the method published by Niazvand et al. [17]. The PLGA/chloroform solution (oil phase) was prepared by dissolving 60 mg of PLGA in 1 mL of chloroform. Then, 6 mg of CUR was added to the solution and sonicated, resulting in a solid/oil emulsion. Thereafter, ethanol and 2% PVA (1 : 1) was added to the emulsion and sonicated for 10 min to produce a solid/oil/weight (s/o/w) emulsion. The s/o/w emulsion was further sonicated and agitated by a magnetic stirrer for 5–6 h to evaporate the solvent (chloroform). The sample was subsequently centrifuged for 10 min at 15,000 g before being rinsed three times using distilled water. The sample was allowed to freeze-dry for 24 h to get a dry powder. The obtained NPs were kept at 4°C.

### 2.4. Characterization of CUR-Loaded PLGA NPs

The surface morphology of CUR-loaded PLGA NPs was investigated with a scanning electron microscope (Zeiss Sigma 500 VP Analytical FE-SEM, Carl Zeiss Germany) and X-ray diffraction (XRD) analysis (model no: 202964, Pananlytical Empyrean company). In addition, their zeta potential and size were detected by Malvern (Malvern Instruments Ltd) following the method by Moaty et al. [18].

### 2.5. Experimental Design

**Figure 1:**
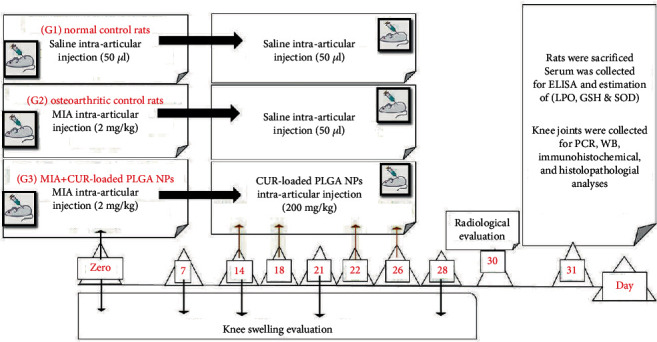
Timeline of the experiment showing the animal groupings, osteoarthritis (OA) induction on day 0, treatment on days 14, 18, 22, and 26, and animal euthanasia on day 31.

As described in Figure 1, the rats were randomly allocated into three groups (*n* = 6). At 0, 14, 18, 22, and 26 days, the normal control group took IAIs of 50 *μ*L of saline into the left side knee joint, whereas the other two groups received IAIs of MIA (2 mg) into the left side knee joints on day 0. Then, rats in the OA control group were given saline IA injections on days 14, 18, 22, and 26, while the treatment group (MIA+CUR-loaded PLGA NPs) received intra-articular injections of CUR-loaded PLGA NPs at a dose of 200 mg/kg. MIA and CUR-loaded PLGA NPs injection doses were based on the previous studies by Ragab et al. [16] and Niazvand et al. [17], respectively.

### 2.6. Knee Diameter Measurement (Swelling)

A manual caliper was used to assess the variations in the anterior-posterior diameter values of the knee joints among all groups [19]. The measurements were obtained on days 0, 7, 14, 21, and 28 after MIA injection.

### 2.7. X-Ray Examination

On day 30 post-MIA injection, animals from all groups were anesthetized using diethyl ether, and both hind limbs were extended and fixed on the table with tape. Radiographs of the left knees (anterior-posterior position) were captured using an X-ray device with a 60 cm focal film distance at 55 kV and 3 mA.

### 2.8. ELISA Evaluation

Inflammatory status was evaluated in all groups by measuring the serum proinflammatory cytokines, tumor necrosis factor-*α* (TNF-*α*) (cat# MBS697379), transforming growth factor-beta (TGF-*β*) (cat # MBS8819920), interleukin- (IL-) 6 (cat# MBS7727039), and IL-1*β* (cat# MBS697409) and the anti-inflammatory cytokine IL-10 (cat# MBS2707969) using MyBioSource Inc., San Diego, CA, USA, following the manufacturer's instructions.

### 2.9. Evaluation of Antioxidant Markers and Oxidative Stress

#### 2.9.1. Determination of Lipid Peroxidation Level

Malondialdehyde (MDA) that was generated through the breakdown of polyunsaturated fatty acids was used as an index for assessment of the extent of lipid peroxidation in serum following the method of Preuss et al. [20].

#### 2.9.2. Determination of Glutathione (GSH) Content and Superoxide Dismutase (SOD) Activity

GSH content in serum was assayed following the procedure of Beutler et al. [21], while the detection of serum SOD relied on the enzyme's capacity to prevent the phenazine methosulphate-mediated decrease of nitroblue tetrazolium dye according to Nishikimi et al. [22].

### 2.10. Quantitative Reverse Transcription Polymerase Chain Reaction (qRT-PCR) Assay

The mRNA levels of nuclear factor-kappa B (NF-*κ*B), type II collagen, and inducible nitric oxide synthase (iNOS) were determined by real-time qRT-PCR, for which the Qiagen tissue extraction kit (Qiagen, USA) was applied for the total RNA isolation. Then, 0.5–2 *μ*g total RNA was applied for cDNA synthesis utilizing a Fermentas kit (USA). Applied Biosystem software version 3.1 (StepOne, USA) was utilized for real-time qPCR amplification and analysis. The qRT-PCR assay was done with primer sets optimized for the annealing temperature. The sequences of the primers are listed in Table 1.

### 2.11. Western Blot Assay

The impact of CUR-loaded PLGA NPs on the protein expression levels of NF-*κ*B50 and NF-*κ*B65 and cleaved caspase-3 was investigated using knee samples maintained at 80°C. Briefly, all samples were homogenized in a radio-immunoprecipitation (RIPA) buffer supplemented with proteinase inhibitors and centrifuged, and the protein concentration was assayed with Bradford assay in the obtained clear supernatant. Proteins (30 mg) were separated on SDS-PAGE, moved to nitrocellulose membranes, and blocked with TRIS using 3% bovine serum albumin and a Tween 20 (TBST) buffer at ambient temperature for 1 hour. Subsequently, membranes were left in the incubator with primary antibodies against NF*κ*Bp50 (Cat# 14-6732-81; eBioscience), NF-*κ*Bp65 (Cat# 14-6731-81, eBioscience), and cleaved caspase-3 (cat # PA5-114687, Thermo Fisher Scientific). Following the washing with TBST, the prepared membranes were left in the incubator with the corresponding secondary antibodies, after which a chemiluminescence kit (BIORAD, USA) was applied. The developed blots were scanned, and image analysis software was utilized to measure the obtained band intensity of the targeted proteins against the control sample after being normalized by beta-actin on a Chemi Doc MP imager.

### 2.12. Histopathological Analysis

The left knee joints were assembled from all groups, followed by fixing in 10% neutral buffered formalin for 48 hours, and then decalcified using 20% EDTA for two weeks. The joints were excised sagittal and processed to get 4–6 *μ*m paraffin-embedded sections. Obtained sections were then stained using hematoxylin and eosin (H&E) before being examined under a light microscope.

### 2.13. Immunohistochemistry Analysis

The sections were stained following the streptavidin-biotin-peroxidase staining method [23]. Paraffin tissue sections (4–6 *μ*m) were deparaffinized in xylene and then rehydrated in ethanol. Endogenous peroxidase and nonspecific binding sites for antibodies were suppressed by treating the sections for 10 min with hydrogen peroxide (0.3%) and for 20 min with 5% normal bovine serum (1 : 5 diluted tris buffer saline [TRIS]) at ambient temperature, respectively. Obtained sections were rinsed with PBS, and then, 10% normal goat serum was applied for 30 min to minimize nonspecific binding. Obtained sections were then incubated in anti MMP13 primary antibody (cat no: GB11247; Servicebio, China) for 1 h, followed by incubation in streptavidin horseradish peroxidase (Dako-K0690) and biotinylated secondary antibody (Dako Universal LSAB Kit) for 15 min and then incubated in 3-diaminobenzidine tetrahydrochloride (Sigma-D5905; Sigma-Aldrich Company Ltd., Gillingham, UK) substrate kit for 10 min to achieve immunolabelling. Afterward, the nuclei were stained using Harry's hematoxylin stain, dehydrated ethanol, cleared in xylene, and then mounted in DPX. Antibody binding was observed with high-power light microscopy.

### 2.14. Statistical Analysis

Statistical analyses were performed using SPSS software version 25.0 (SPSS Inc., Chicago, IL, USA). All results are presented as means (M) ± standard errors of means (SEMs), with *P* < 0.05 meaning a statistically significant difference.

## 3. Results

### 3.1. Characterization of CUR-Loaded PLGA NPs

**Figure 2:**
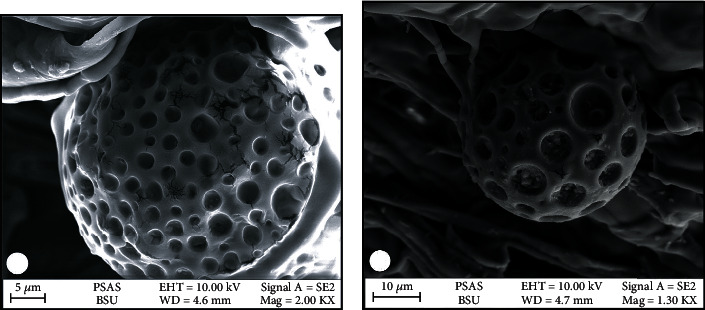
Scanning electron micrograph showing (a) PLGA and (b) CUR-loaded PLGA NPs.

**Figure 3:**
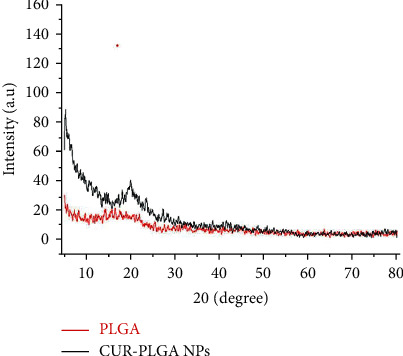
XRD of PLGA and CUR-loaded PLGA NPs.

**Figure 4:**
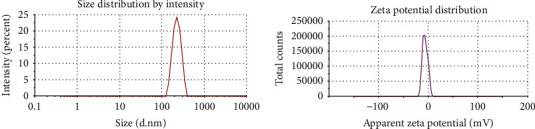
Illustration of the (a) size and (b) zeta potential of CUR-loaded PLGA nanoparticles.

In this study, the prepared CUR-loaded PLGA NPs were shown using a scanning electron microscope micrograph (Figure 2), which were spherically shaped. Meanwhile, the XRD pattern of CUR-loaded PLGA NPs (Figure 3) showed an absence of marked crystalline domains in CUR-loaded PLGA NPs, implying that these NPs were in the disordered-crystalline phase or the amorphous or the solid-state solubilized form in the polymer matrix. Further, the average size (Figure 4(a)) and zeta potential (Figure 4(b)) of the CUR-loaded PLGA NPs were 265.2 nm and −6.86 mV, respectively.

### 3.2. Effect of CUR-Loaded PLGA NPs on Swelling (Knee Diameter Measurements)

**Figure 5:**
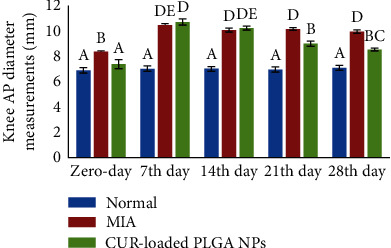
Knee anterior-posterior diameter measurements in the normal control, monosodium iodoacetate (MIA), and MIA+CUR-loaded PLGA NP groups. At each period, the means, which have different symbols (letters), are significantly different at *P* < 0.05.

As shown in Figure 5, MIA induced an increase in the knee diameters of the OA control and treatment groups when compared to the measurements before the injection. Nevertheless, two weeks of CUR-loaded PLGA NP IAIs markedly reduced swelling in the left knee joints.

### 3.3. Effect of CUR-Loaded PLGA NPs Using X-Ray Imaging

**Figure 6:**
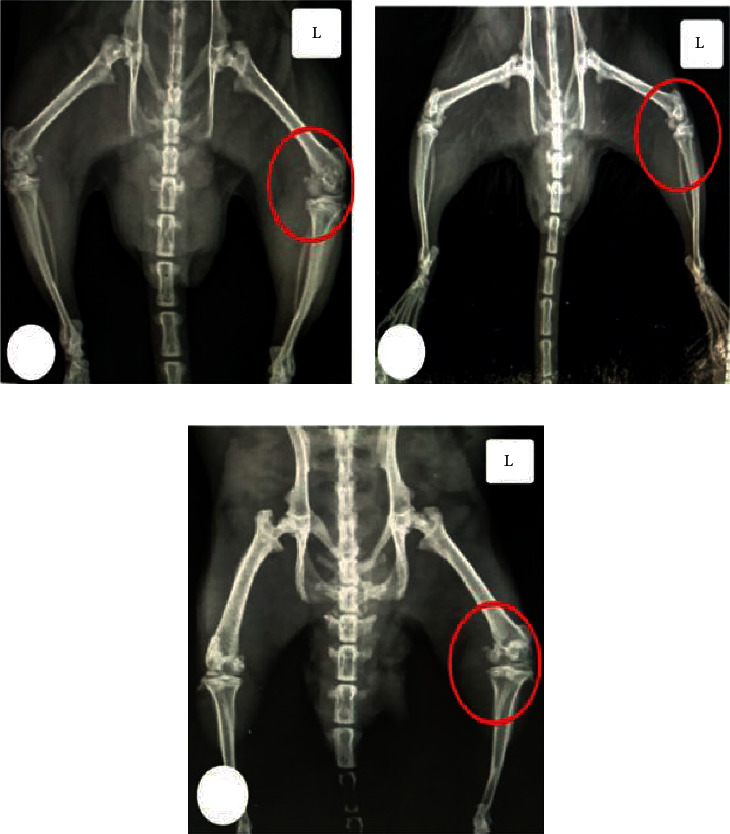
X-ray image showing the left knee joints (L) of all groups: (a) the normal knee joints; (b) MIA-treated knee joints depicting radiographic alternations such as erosion of the cartilage surface, osteophytes, and joint space narrowing; and (c) MIA+CUR-loaded PLGA NP-treated group, with joints nearly similar to those of the normal control.

Radiographic detection was carried out to observe changes in the knee joints after CUR-loaded PLGA NP treatment. Compared to that of the normal control group (Figure 6(a)), knee joints in the OA group (Figure 6(b)) had a narrow joint space and deformed articular surface. Meanwhile, the CUR-loaded PLGA NP treatment considerably hindered MIA-induced knee OA progression and attenuated joint destruction, in that the joints of the treated group (Figure 6(c)) showed a little degree of narrowing, and no obvious sclerosis and osteophyte formation was detected.

### 3.4. Effect of CUR-Loaded PLGA NPs on the Serum Levels of TNF-*ɑ*, IL-1*β*, IL-6, TGF-*β*, and IL-10

The MIA-treated group (Table 2) showed a significant increase in serum TNF-*ɑ*, IL-6, IL-1*β*, and TGF-*β* levels and a decrease in IL-10 levels compared to those of the normal control group (*P* < 0.05). Meanwhile, the OA knee joints from the treatment group showed a marked reduction in TNF-*ɑ*, IL-6, IL-1*β*, and TGF-*β* levels, along with an elevation in IL-10 levels as compared to those of the OA group.

### 3.5. Effect of CUR-Loaded PLGA NPs on Lipid Peroxidation and Antioxidant Status

The enhanced level of serum lipid peroxidation product (MDA) in MIA-treated knee joints was accompanied by a substantial reduction in GSH level and SOD activity. Meanwhile, CUR-loaded PLGA NP IAIs significantly lowered the level of MDA and boosted the GSH content and SOD activity (Table 3).

### 3.6. Effect of CUR-Loaded PLGA NPs on NF-*κ*B, iNOS, and Type II Collagen mRNA Expression

OA knee joints showed a marked (*P* < 0.05) elevation in the mRNA expression levels of NF-*κ*B and iNOS, along with a significant decline in type II collagen compared with those of the normal group (Table 4). Moreover, OA knee joints treated with CUR exhibited a significant (*P* < 0.05) downregulation in NF-*κ*B and iNOS levels and inhibited the loss of type II collagen mRNA expression level in comparison with that in OA control rats.

### 3.7. Effect of CUR-Loaded PLGA NPs on the Protein Expression Levels of NF-*κ*B p50 and NF-*κ*B p65

**Figure 7:**
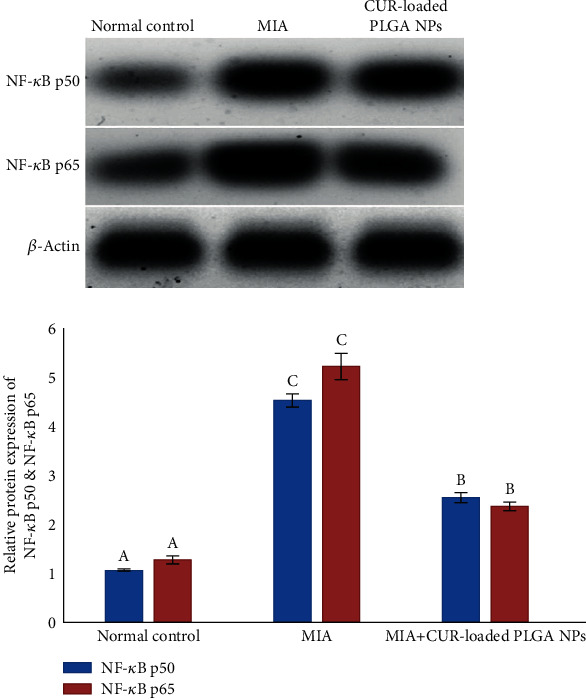
Effect of CUR-loaded PLGA NPs on the relative protein expression levels of NF-*κ*B p50 and NF-*κ*B p65 of MIA-induced OA rats. Means, which have different symbols (letters), are significantly different at *P* < 0.05.

Western blot analysis shows that the protein expression levels of NF-*κ*B p50 and NF-*κ*B p65 (Figures 7) were elevated post-MIA administration when compared with the normal control. However, CUR-loaded PLGA NP treatment significantly diminished the protein expression of NF-*κ*B p50 and NF-*κ*B p65 in the OA knee joints as compared to the MIA group.

### 3.8. Effect of CUR-Loaded PLGA NPs on Cleaved Caspase-3 Protein Expression

**Figure 8:**
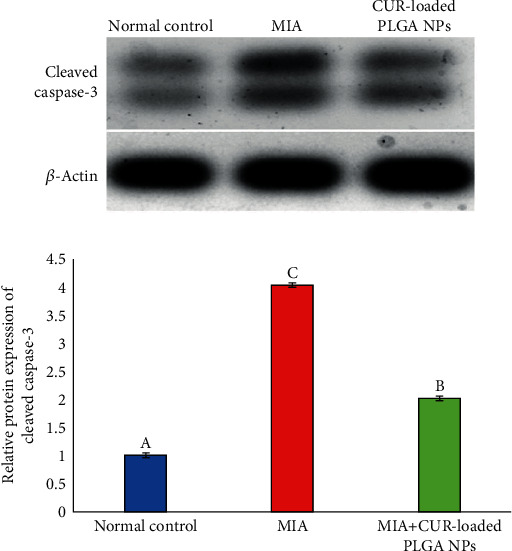
Effect of CUR-loaded PLGA NPs on the relative protein expression level of cleaved caspase-3 of MIA-induced OA rats. Means, which have different symbols (letters), are significantly different at *P* < 0.05.

The protein level of cleaved caspase-3 (Figure 8) in the knee joint was estimated with western blotting. OA rats demonstrated a marked increase in cleaved caspase-3 level relative to those in the normal control group, while OA rats injected with CUR-loaded PLGA NPs revealed a marked downregulation in the cleaved caspase-3 level compared with those in the OA control group.

### 3.9. Effect of CUR-Loaded PLGA NPs on the Histopathological Evaluation

**Figure 9:**
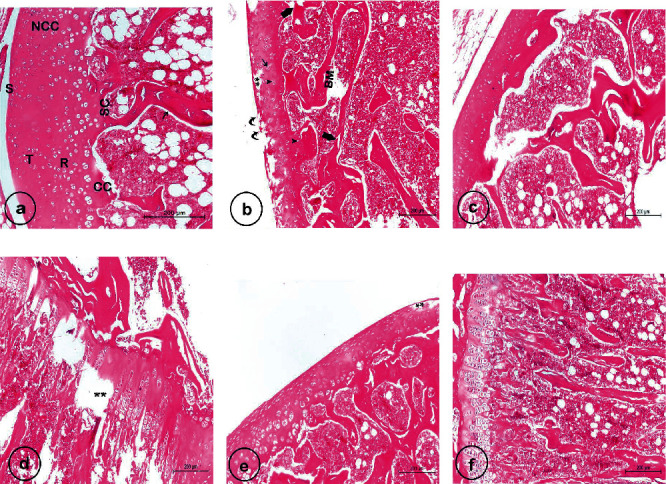
Photomicrographs of hematoxylin and eosin- (H&E-) stained sections of the left knee joints. (a) shows the normal control group with the normal architecture of the articular cartilage that consists of a noncalcified (NCC) region, which is arranged into superficial (S), transitional (T), and radial (R) zones and calcified (CC) region with a clear intact tidemark in between (scale bar = 200 *μ*m). It also shows subchondral bone (SC) with well-oriented bony trabeculae (arrow). (b)–(d) show the MIA-treated group (osteoarthritic rats), wherein (b) depicts fissures, surface fibrillation (curved arrows), chondrocytes with hyperchromatic nuclei (arrowheads), chondrocytes clusters (thin arrow), marked loss of (matrix and chondrocytes), and degenerated and disorganized bone trabeculae (thick arrows); (c) shows a decrease in articular cartilage thickness, unclear tidemark, an abnormal subchondral with an increase in trabecular thickness, and bone marrow space (BM) containing fewer hematopoietic cells; and (d) shows degeneration (asterisk) and heterogeneous distribution of chondrocytes in the growth plate. (e) and (f) show the treatment group (MIA+CUR-loaded PLGA NPs), wherein (e) displays a marked restoration of the normal structure of articular cartilage with an intact surface, except for some damaged parts (^∗∗^) and a few hyperchromatic nuclei, an increase in cellularity, a partial improvement in tidemark integrity, and nearly normal bone marrow space (BM) relative to the MIA group (scale bar = 200 *μ*m), and (f) demonstrates neatly and properly aligned and oriented chondrocytes of the growth plate (scale bar = 200 *μ*m).

H&E sections of the articular cartilage showed that chondrocytes in the normal control group were placed neatly, and the dyeing was uniform (Figure 9(a)). In the OA control group, obtained sections had a myriad of pathological alternations such as cracks, fibrillation, disorderly arranged cells, empty lacunae, hyperchromatic nuclei, and a magnificent reduction in the number of chondrocytes (Figures 9(b)–9(d)). In contrast, the MIA+CUR-loaded PLGA NP group (Figures 9(e)) showed a notable decrease in the severity of cartilage degradation, as the CUR treatment offered effective protection against OA progression. MIA+CUR-loaded PLGA NP-treated cartilage appeared to have a smooth surface, orderly arranged chondrocytes, less loss of cells, and intact subchondral bone compared to those of MIA-treated cartilages without treatment.

### 3.10. Effect of CUR-Loaded PLGA NPs on the Matrix Metalloproteinase-13 (MMP-13) Expression

**Figure 10:**
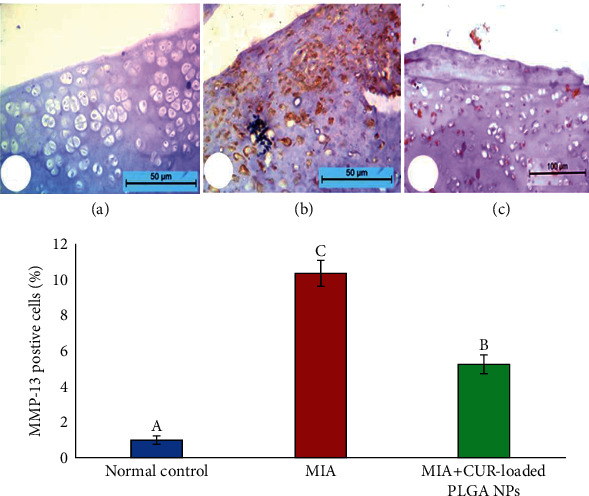
Photomicrograph showing immunostaining results of matrix metalloproteinase-1 (MMP-13) expression in cartilage tissue in the (a) normal control group, (b) MIA-treated group, and (c) MIA+CUR-loaded PLGA NP group. Stained cells (brown) were counted, and the percentage of positive cells is expressed as mean ± standard error of the mean (SEM), where *n* = 6 and *P* < 0.05.

MMP-13, a key catabolic enzyme, was immunohistochemically stained in the chondrocytes of the articular cartilage to assess its protein expression. When compared with the normal control group, which almost had no positive staining for MMP13 (Figure 10(a)), MIA induced an elevation in MMP-13 content in the articular cartilage of the OA control group (Figure 10(b)). However, sections (Figure 10(c)) showed that the MMP-13 content was notably diminished in the articular cartilage of the MIA+CUR-loaded PLGA NP group as compared to that of the OA control group.

Additionally, our results (Figure 10) demonstrate an increase in cell staining positive for MMP-13 in the articular cartilage following MIA injection compared with that of the normal control group (10.39 ± 0.22 vs. 1.01 ± 0.23, *P* < 0.05, respectively). However, osteoarthritic rats treated with CUR-loaded PLGA NPs exhibited a marked reduction in MMP-13-positive chondrocytes compared with that of the MIA-treated group without any treatment (5.26 ± 0.74 vs. 10.39 ± 0.22, *P* < 0.05).

## 4. Discussion

Currently, evidence shows that there is a significant correlation between the incidence and progression of OA and inflammation, oxidative stress, and excessive catabolic activity [24].

**Figure 11:**
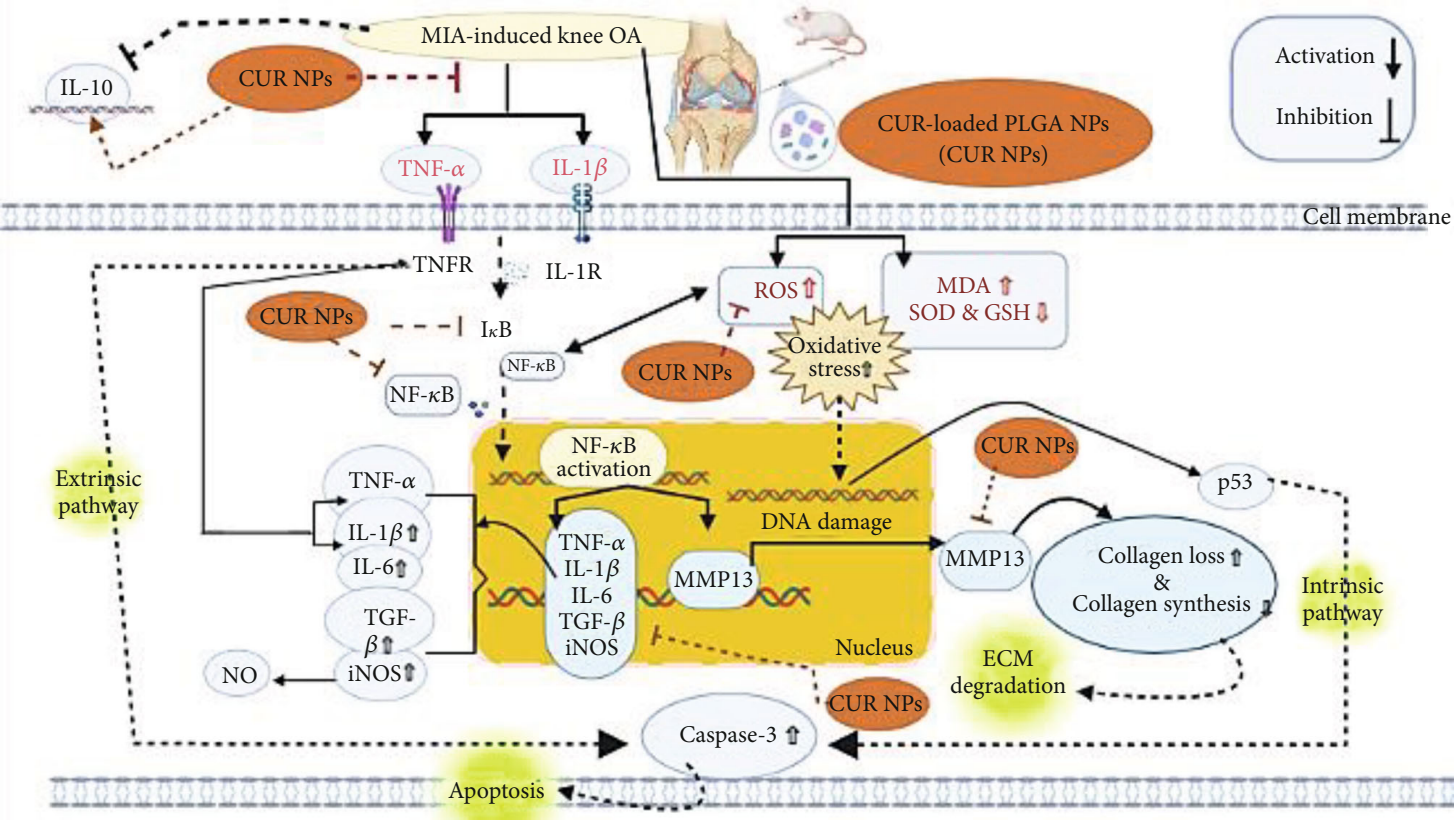
Therapeutic effect of CUR-loaded PLGA NPs against cartilage damage due to inflammation, extracellular matrix (ECM) degradation, and apoptosis in MIA-induced knee OA.

In the current work, we utilized MIA to generate histological and biochemical changes in the articular cartilage that resemble OA conditions in humans [25]. Moreover, in search of inexpensive and beneficial treatments against OA, we prepared CUR-loaded PLGA NPs and investigated their potency against inflammatory mediators, oxidative stress, and chondrocyte apoptosis in MIA-induced OA in a rat model (Figure 11).

The sizes of the CUR-loaded PLGA NP detected in our study were smaller than 300 nm and were consistent with those reported by Gonzales et al. [26]. While the XRD pattern in our study displayed no typical CUR peaks when entrapped in NPs, Khan et al. [27] elucidated that the absence of any noticeable crystalline domains of CUR implies that CUR loaded on PLGA NPs is in the disordered-crystalline phase or the amorphous or the solid-state solubilized form in the polymer matrix. This disordered-crystalline phase, or CUR, inside the polymeric matrix, allows for a controlled release of the encapsulated drug from the NPs.

Following Yabas et al. [28], our radiographic results revealed that CUR NP intra-articular administration markedly lessened the MIA-induced radiographic abnormalities in the knee joints of the treated rats represented by normal joint space and smooth surface of articular cartilage.

The NF-*κ*B signaling pathway is claimed to be one of the main signaling pathways that promote the progression of OA [29]. Although NF-*κ*B stays inactive in the cytoplasm under normal conditions, upon adequate stimulation, e.g., by inflammatory cytokines or the inflammatory microenvironment, I*κ*B kinase (IKK) activity phosphorylates I*κ*B proteins and causes their degeneration, which allows free NF-*κ*B complexes to translocate from the cytoplasm into the nucleus and stimulate a variety of inflammation-related genes. Furthermore, it triggers extracellular matrix degradation, chondrocyte apoptosis, pannus formation, and, eventually, pathological cartilage destruction (Figure 11) [5, 30].

Alternatively, various studies assumed that CUR hinders OA-related inflammation by blocking the NF-*κ*B signaling pathway and preventing chondrocyte apoptosis [31, 32]. As a result, OA-related inflammation is suppressed and progression is slowed [11, 33, 34].

Subsequently, our study discussed the effect of CUR-loaded PLGA NP IAIs on NF-*κ*B gene and NF-*κ*B-regulated genes participating in inflammation, such as TNF-*α*, IL-6, IL-1*β*, and TGF-*β*. Our data in harmony with Alhusaini et al. [30] propose that CUR administration blocked NF-*κ*B activation by inhibiting I*κ*B degradation and phosphorylation and suppressing the translocation of NF-*κ*B into the nucleus, thereby impeding the inflammatory response of the cells.

Additionally, in agreement with [35], our presented data revealed that CUR considerably boosted the serum levels of IL-10, a robust anti-inflammatory immunosuppressive and chondroprotective cytokine, indicating its potent anti-inflammatory capacity.

Furthermore, CUR-loaded PLGA NP treatment during OA inhibited the expression of iNOS, which is an inflammation-induced enzyme that catalyzes the production of the proinflammatory mediator nitric oxide (NO), further demonstrating its anti-inflammatory effects. Several *in vivo* and *in vitro* studies [36, 37] have shown that CUR treatment reduces iNOS production in various inflammatory diseases. Most recently, Cheragh-Birjandi et al. [37] postulated that CUR administration can regulate NO levels by suppressing the activation of the N-terminal kinase (JNK), p38, and NF-*κ*B pathways.

NF-*κ*B pathway activation not only upregulates proinflammatory mediators but additionally mediates the chondrocyte activation triggered by the released extracellular matrix (ECM) products, e.g., fibronectin fragments, which in turn promote the expression of a variety of matrix-degrading enzymes, including metalloproteinases (MMPs) [38–40]. In the early stage of OA, high upregulation of MMP-13 expression, a prominent catabolic enzyme, leads to severe deterioration of cartilage as it induces type II collagen, which is a reason for nearly 90% of the ECM. The loss of type II collagen is a critical stage that determines the irreversible progression of OA (Figure 11) [41]. Therefore, this study explored MMP-13 and collagen type II expression levels as indicators of the anticatabolic activity of CUR NPs in the OA knee joint.

Herein, IAIs of CUR NPs prevented OA exacerbation by diminishing MMP-13 expression [42] and the degradation of type II collagen [6]. Kumar et al. [43] posited that CUR possesses a suppressing effect on MMPs and variable cellular signaling pathways, e.g., Janus kinase STAT and NF-*κ*B/mitogen-activated protein kinase/phosphoinositide 3-kinase, which eventually ameliorate OA and prevent the further damage of the cartilage.

Further, the effect of CUR treatment on cell death in knee OA has also been elucidated. Although apoptosis is a crucial process in keeping the homeostasis of several tissues, the high rate of chondrocyte death is a well-known pathological feature of OA [44]. MIA-induced TNF-*α* activates the tumor necrosis factor receptor (TNFR) or death receptors, eventually triggering the extrinsic pathway of apoptosis (Figure 11). While Ding et al. [45] postulated that in MIA-induced OA, the intrinsic pathway of apoptosis can be triggered by reactive oxygen species (ROS). ROS accumulation could induce oxidative stress, thus altering mitochondrial function and promoting the excretion of cytochrome c and activation of proapoptotic factors, e.g., caspase-3 [44, 46]. Moreover, mitochondrial impairment was shown to enhance the response to cytokine-induced chondrocyte inflammation by producing ROS and activating NF-*κ*B [47].

Conversely, CUR has been shown to restore mitochondrial functions, scavenge free radicals and ROS [48, 49], and suppress lipid peroxidation [50]. CUR also enhances the activity of other antioxidants such as SOD, catalase, GSH, and glutathione peroxidase in various diseases [30, 51]. Based on these studies, we scrutinized the role of CUR-loaded PLGA NPs in suppressing the activation of the apoptosis mediators related to OA. Our data confirmed the antioxidant efficacy of CUR NPS as they considerably averted the increase in MDA lipid peroxidation product and enhanced the antioxidant status (GSH concentration and SOD activity). Moreover, consistent with the results of Buhrman et al. [52], our results revealed the antiapoptotic efficacy of CUR NPS, as they notably hindered upregulation in caspase-3 levels. The antioxidant, anticatabolic, and antiapoptotic potency can be mostly attributed to the hydroxyl, methoxy, *α*, *β*-unsaturated carbonyl, or diketone groups present in CUR [53].

MIA-induced knee OA inflammation led to severe histopathological alterations in the architecture of the articular cartilage, such as clefts, disorderly arranged cells, hyperchromatic, and a significant reduction in chondrocyte number [31]. Whereas CUR-loaded PLGA NP treatment did not affect the regeneration of the cells, it halted OA progression by reducing inflammation and chondrocytes apoptosis which is depicted by milder tissue defects and pronounced articular cartilage and subchondral bone integrity. Our findings are consistent with Yabas et al. [28], which revealed that CUR lessened OA severity by restoring the architecture of the knee joint. Wang et al. [54] postulated that CUR moderately enhanced the integrity of the articular cartilage by blocking the NF-*κ*B/hypoxia-inducible factor 2*α* signaling pathway.

Therefore, current findings suggest that OA-related deteriorations and progression are halted due to the ability of CUR-loaded PLGA NPs to modulate various mediators such as NF-*κ*B, MMP-13, and oxidative stress.

## 5. Conclusion

Overall, this study demonstrates that intra-articular treatment with CUR-loaded PLGA nanoparticles is a compelling candidate for improving joint health in knee OA due to their anti-inflammatory, anticatabolic, and antioxidant characteristics. However, for clinical applications, further research studies with a longer treatment period and bigger sample size should be carried out to explore its underlying mechanisms.

## Figures and Tables

**Table 1:** Primer sequences for NF-*κ*B, iNOS, and type II collagen mRNA.

Target gene	Primer sequence
NF-*κ*B	Forward primer: 5′-CATTGAGGTGTATTTCACGG-3′ Reverse primer: 5′-GGCAAGTGGCCATTGTGTTC-3′
iNOS	Forward primer: 5′-GACCAGAAACTGTCTCACCTG-3′ Reverse primer: 5′-CGAACATCGAACGTCTCACA-3′
Type II collagen	Forward primer: 5′-GAGTGGAAGAGCGGAGACTACTG-3′ Reverse primer: 5′-CTCCATGTTGCAGAAGACTTTCA-3′
Beta-actin	Forward primer: 5′-TGTTTGAGACCTTCAACACC-3′ Reverse primer: 5′-CGCTCATTGCCGATAGTGAT-3′

There were six samples in each group, and data are described as means ± SEM. For each parameter, means (which have different superscript symbols) are statistically significant at *P* < 0.05.

**Table 2:** Effect of CUR-loaded PLGA NPs on the serum levels of TNF-*α*, IL-1*β*, IL-6, TGF-*β*, and IL-10 in MIA-induced OA in rats.

Groups	Parameters				
	TNF-*α* (pg/mL)	IL-1*β* (pg/mL)	IL-6 (pg/mL)	TGF-*β* (pg/mL)	IL-10 (pg/mL)
Normal control	20.22 ± 1.1^a^	47.05 ± 2.96^a^	62.78 ± 2.92^a^	115.36 ± 1.36^a^	324.67 ± 10.28^c^
MIA	261.19 ± 3.47^c^	136.07 ± 4.17^c^	188.9 ± 1.1^c^	260.55 ± 5.26^c^	122.87 ± 4.04^a^
MIA+CUR-loaded PLGA NPs	57.02 ± 1.23^b^	65.57 ± 2.92^b^	94.88 ± 2.62^b^	146.25 ± 5.61^b^	283.57 ± 4.36^b^

There were six samples in each group, and data are described as means ± SEM. For each parameter, means, which have different superscript symbols, are statistically significant at *P* < 0.05.

**Table 3:** Effect of CUR-loaded PLGA NPs on the serum MDA and GSH levels and SOD activity in MIA-induced OA rats.

Groups	Parameters		
	MDA (nmol/mL)	GSH (mg/dL)	SOD (U/mL)
Normal control	0.84 ± 0.07^a^	210.70 ± 10.40^c^	263.11 ± 11.71^c^
MIA	2.095 ± 0.37^c^	9.2213 ± 0.66^a^	55.09 ± 9.97^a^
MIA+CUR-loaded PLAGA NPs	0.89 ± 0.11^b^	41.46 ± 5.99^b^	111.16 ± 6.28^b^

There were six samples in each group, and data are described as means ± SEM. For each parameter, means, which have different superscript symbols, are statistically significant at *P* < 0.05.

**Table 4:** Effect of CUR-loaded PLGA NPs on mRNA relative expression of NF-*κ*B, iNOS, and type II collagen of MIA-induced OA rats.

Groups	Parameters		
	NF-*κ*B	iNOS	Type II collagen
Normal control	0.094 ± 0.0095^a^	1 ± 0.0106^a^	1.05 ± 0.01^c^
MIA	6.72 ± 0.33^c^	5.64 ± 0.28^c^	0.25 ± 0.06^a^
MIA+CUR-loaded PLGA NPs	1.94 ± 0.16^b^	2.13 ± 0.18^b^	0.5 ± 0.04^b^

There were six samples in each group, and data are described as means ± SEM. For each parameter, means, which have different superscript symbols, are statistically significant at *P* < 0.05.

## Data Availability

The authors confirmed that all data generated or analyzed during this study are included in this published article.

## Abbreviations

Cur: Curcumin
CUR-loaded PLGA NPs: Curcumin-loaded polylactic-co-glycolic acid nanoparticles
ECM: Extracellular matrix
GSH: Reduced glutathione
IAIs: Intra-articular injections
IFN-*γ*: Interferon-*γ*
IL: Interleukin
iNOS: Inducible nitric oxide synthase
MDA: Malondialdehyde
MIA: Monosodium iodoacetate
MMP-13: Matrix metalloproteinase-1
NF-*κ*B: Nuclear factor-kappa B
NO: Nitric oxide
NPs: Nanoparticles
NSAIDs: Nonsteroidal anti-inflammatory drugs
OA: Osteoarthritis
PLGA: Polylactic-co-glycolic acid
PVA: Polyvinyl alcohol
RIPA: Radio-immunoprecipitation assay
ROS: Reactive oxygen species
SDS-PAGE: Sodium dodecyl sulfate-polyacrylamide gel electrophoresis
STAT: Janus kinase signal transduction and activation transcription
TBA: Thiobarbituric acid
TGF-*β*: Transforming growth factor-beta
TNFR: Tumor necrosis factor receptors
TNF-*α*: Tumor necrosis factor-*α*.
